# Radical Generation from the Gas-Phase Activation of Ionized Lipid Ozonides

**DOI:** 10.1007/s13361-017-1649-4

**Published:** 2017-05-08

**Authors:** Shane R. Ellis, Huong T. Pham, Marc in het Panhuis, Adam J. Trevitt, Todd W. Mitchell, Stephen J. Blanksby

**Affiliations:** 1 M4I, The Maastricht Multimodal Molecular Imaging Institute, University of Maastricht, 6229 ER Maastricht, The Netherlands; 2 School of Chemistry, University of Wollongong, Wollongong, NSW 2522 Australia; 3 Boreal Ecosystem Research Initiative (BERI), Environmental Science, Memorial University, Grenfell Campus, Corner Brook, NL A2H 5G4 Canada; 4 School of Medicine, University of Wollongong, Wollongong, NSW 2522 Australia; 5 Central Analytical Research Facility, Institute for Future Environments, Queensland University of Technology, Brisbane, QLD 4001 Australia

**Keywords:** Ozonolysis, Secondary ozonide, Electrospray ionization, Free radical, Collision-induced dissociation, Photo dissociation, Aerosols

## Abstract

Reaction products from the ozonolysis of unsaturated lipids at gas–liquid interfaces have the potential to significantly influence the chemical and physical properties of organic aerosols in the atmosphere. In this study, the gas-phase dissociation behavior of lipid secondary ozonides is investigated using ion-trap mass spectrometry. Secondary ozonides were formed by reaction between a thin film of unsaturated lipids (fatty acid methyl esters or phospholipids) with ozone before being transferred to the gas phase as [M + Na]^+^ ions by electrospray ionization. Activation of the ionized ozonides was performed by either energetic collisions with helium buffer-gas or laser photolysis, with both processes yielding similar product distributions. Products arising from the decomposition of the ozonides were characterized by their mass-to-charge ratio and subsequent ion-molecule reactions. Product assignments were rationalized as arising from initial homolysis of the ozonide oxygen–oxygen bond with subsequent decomposition of the nascent biradical intermediate. In addition to classic aldehyde and carbonyl oxide-type fragments, carbon-centered radicals were identified with a number of decomposition pathways that indicated facile unimolecular radical migration. These findings reveal that photoactivation of secondary ozonides formed by the reaction of aerosol-bound lipids with tropospheric ozone may initiate radical-mediated chemistry within the particle resulting in surface modification.

**Electronic supplementary material:**

The online version of this article (doi:10.1007/s13361-017-1649-4) contains supplementary material, which is available to authorized users.

**Graphical Abstract:**
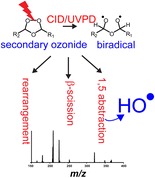
ᅟ

## Introduction

Large amounts (Tg year^–1^) of short-chain alkenes, in the form of volatile organic compounds, are emitted into the atmosphere every year from a variety of biogenic and anthropogenic sources [1–3]. Less volatile long-chain molecules such as fatty acids and other lipids are also emitted into the atmosphere through a variety of natural and anthropogenic processes and are well known to be components of atmospheric aerosols [2–6]. Lipids are important components of all cell membranes and thus form a significant part of the organic layer on the surface of sea water following the decomposition of marine organisms [7–12]. Wave action on the ocean surface leads to the formation of sea salt based aerosols onto which organic material from the surface layer—including lipids derived from marine life—become bound. Once formed, marine aerosols are exposed to oxidizing species in the atmosphere. The oxidation of organic matter on aerosol particles will alter their hygroscopic properties and thus impact mechanisms of cloud condensation [9, 10, 13, 14].

**Scheme 1:**
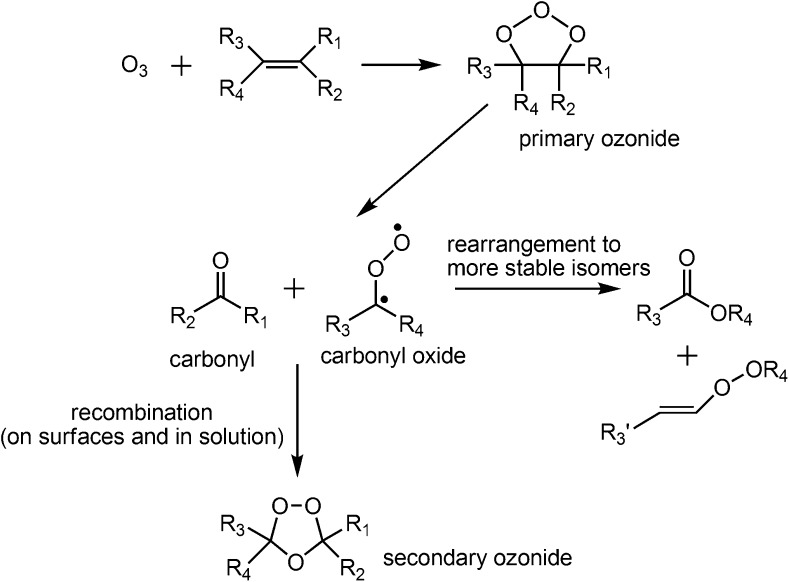
Criegee mechanism of alkene ozonolysis [15]

Ozone is one of the important atmospheric oxidants, particularly for unsaturated species, and can react with such molecules either in the gas-phase or on surfaces (e.g., aerosol particles). The accepted mechanism for ozonolysis was proposed by Criegee in the 1950s and is summarized in Scheme 1 [15]. Briefly, ozone first adds across a carbon–carbon double bond to form an unstable primary ozonide (or 1,2,3-trioxolane) that readily decomposes to form an aldehyde and a vibrationally excited carbonyl oxide (sometimes referred to as the Criegee intermediate). The initial addition of ozone to a double bond to form the primary ozonide (Scheme 1) has been computed to be exothermic by ~55 kcal mol^–1^ in the case of ethene [16]. On surfaces and in solution where a solvent cage exists, the aldehyde and carbonyl oxide intermediates are held in close proximity. Under these conditions, the reactive intermediates can recombine to form a more stable secondary ozonide (or 1,2,4-trioxolane) (Scheme 1). Ozonolysis on surfaces such as aerosol particles also facilitates the formation of stable secondary ozonides by providing alternative pathways for the energy from the initial ozone addition to be dispersed. Karagulian et al. [17] have observed the formation of secondary ozonides from unsaturated lipids at the surfaces of model sea salt aerosol, whereas we have observed secondary ozonide formation on various surfaces following ambient ozonolysis of unsaturated phospholipids [18].

In the gas-phase, however, the aldehyde and carbonyl oxide intermediates can become rapidly separated, allowing for comparatively little recombination. Furthermore, in the absence of collisional partners, relaxation of energetically excited carbonyl oxide intermediates is slow, thus allowing for unimolecular rearrangement or decomposition [3]. The formation of stable secondary ozonides is also partly determined by the carbon chain length of the reacting alkene. For example, in larger molecules such as lipids, the excess energy can be redistributed amongst many internal degrees of freedom, making formation of a stable secondary ozonide more probable.

While it is accepted that tropospheric ozone reacts with organics adsorbed onto aerosol particles, and that such reactions can lead to the formation of stable secondary ozonides, the fate of these secondary ozonides in the atmosphere is not as well understood [9, 10, 17, 19]. It is possible that activation of secondary ozonides, either thermally or by photolysis, can facilitate their decomposition into a number of lower mass components. Several studies have investigated the thermal decomposition of secondary ozonides in both the gas-phase and solution-phase, and in all cases aldehydes and carboxylic acids are observed as major products [20–25]. Studies by Hull et al. [22] and Khachatryan et al. [23] have provided evidence for a radical mechanism leading to the formation of aldehydes and carboxylic acids. This involves initial homolysis of the oxygen–oxygen bond to produce a biradical followed by rearrangement and dissociation pathways to give an aldehyde and carboxylic acid. The intermediacy of the biradical is also consistent with a variety of other products observed upon ozonide activation. While it is well known that ozonolysis of alkenes produces hydroxyl radicals [26–30], work by Pryor and co-workers investigating the thermal decomposition of the secondary ozonide of allylbenzene found evidence for the formation of carbon- and oxygen-centered radicals [21]. UV-photolysis of secondary ozonides has also been reported with several studies observing spectroscopic signatures consistent with products of biradical decomposition [17, 24, 31–33]. While prior studies have found evidence for radical formation following secondary ozonide activation, the identity of the radicals themselves is most often not determined.

Mass spectrometry is a powerful tool for observing and studying the structure of reactive intermediates in the gas-phase. Using this approach, Harrison and Murphy [34] studied the fragments arising from collision-induced dissociation (CID) of phospholipid secondary ozonides. Along with the characteristic aldehyde and carboxylic acid fragments (or isomers thereof), ions with mass suggestive of radical ions were observed. These were assigned as carbon-centered radicals formed from the biradical by a β-scission mechanism; however, the structures of the radicals were not investigated further. In the present study, we have probed the intrinsic unimolecular decomposition of lipid secondary ozonides analogous to those likely to be formed on marine aerosols. This has been undertaken by electrospray ionization tandem mass spectrometry (MS/MS), where surface-formed secondary ozonides have been gently transferred to the gas-phase for subsequent activation. The molecular structure of radical products formed by activation has been studied using multi-stage tandem mass spectrometry (MS^n^) and ion-molecule reactions. These data are compared with decomposition products (including radicals) formed by photodissociation (PD) using wavelengths within the actinic window. These investigations support the possibility of unimolecular decomposition of secondary ozonides formed from aerosol lipids as being a source of radicals in the atmosphere.

## Experimental

### Materials

All phospholipids standards were purchased from Avanti Polar Lipids (Alabaster, AL, USA). Fatty acids were purchased from Nu-Chek Prep (Elysian, MN, USA) with the exception of partially deuterated oleic acid (11,11,12,12,13,13,14,14,15,15,16,16,17,17,18,18,18-*D*_17_-9*Z*-octadecenoic acid, 99%), which was purchased from Cayman Chemical (Michigan, MI, USA). Methanol, chloroform, and pentane (all HPLC grade) were purchased from Crown Scientific (Sydney, NSW, Australia). Sodium acetate was purchased from Ajax Chemicals (Sydney, Australia). Boron trifluoride was purchased from Sigma Aldrich (Castle Hill, NSW, Australia). All compounds were used without further purification.

### Fatty Acid Methyl Ester Preparation

Approximately 1 mg of each fatty acid standard was dissolved in 1 mL of 10% boron trifluoride in methanol and stirred for 20 min at room temperature. Water (0.5 mL) and pentane (1 mL) were then sequentially added to the solution to separate aqueous and organic components. The organic layer containing the fatty acid methyl ester (FAME) component was then removed from the organic layer at a concentration of approximately 3 mM in pentane.

### Ozonide Preparation

Ozonide preparation was modeled on the method reported by Harrison and Murphy [34]. First, 20–50 nmol of lipid present as 0.2–3.0 mM solutions in either methanol, 2:1 methanol:chloroform (v/v) (phospholipids), or pentane (fatty acid methyl esters) was deposited into a 10 mL glass vial and the solvent removed under a stream of dry nitrogen. The vial containing the dried lipid film was placed in a Drechsel bottle connected to an ozone generator (HC-30 ozone generator; Ozone Solutions, Sioux Center, IA, USA). Oxygen (backing pressure 140 kPa and a flow of 100–150 mL min^–1^) was passed through the ozone generator and directed into the glass vial. The power output of the ozone generator was 30 (arbitrary units) producing an estimated 15% w/w ozone in oxygen. Thus produced ozone was passed over the lipid film for 2–5 min, after which time the ozone generator was turned off and the system flushed with oxygen for 10 min. During this procedure, excess ozone exiting the Drechsel bottle was destroyed by bubbling through an aqueous solution of sodium thiosulfate. Sodium iodide and Vitex (a starch-based indicator) were also present in the solution to provide a rapid visual indication if the sodium thiosulfate was exhausted [35]. The vial was then removed and lipids and their oxidation products dissolved by addition of ~1.5–2.0 mL 2:1 methanol:chloroform (v/v) giving a final lipid concentration of 10–25 μM. Methanolic sodium acetate was then added to a final salt concentration of 50 μM. This solution was then analyzed by mass spectrometry.

### Electrospray Ionization-Mass Spectrometry

Electrospray Ionization-Mass Spectrometry (ESI-MS) was performed on a Thermo Finnigan LTQ mass spectrometer (now Thermo Fisher Scientific, San Jose, CA, USA) operating Xcalibur 2.0 software. Samples were infused at a flow rate of 3–5 μL min^–1^ with a spray voltage of +4 kV. The automatic tune function was used to optimize the ion optics for the detection of secondary ozonides of each lipid class. The capillary temperature was typically 200 °C and typical tube lens voltages were +235 V for phospholipid ions and +65 V for fatty acid methyl esters. For CID, ions were isolated with an isolation window of 2–3 Th and a resonant excitation was applied using a normalized collision energy [36] between 20% and 30% for a period of 30 ms. In some instances, where isolation of individual isotopologues was difficult with a single isolation step, ions were isolated using a nested isolation procedure in which isolation was repeated three times using a sequence of isolation widths of 5, 2, and 3–5 Th.

For ion-molecule reactions involving background oxygen, the radical precursor was first generated by CID of the corresponding ozonide and then isolated in the ion trap without additional collisional activation (with CE = 0). The reaction time with background oxygen was controlled by adjusting the ion activation time to between 500 and 7000 ms at the relevant step of the MS^n^ experiment. Oxygen concentrations within the instrument under normal operating conditions (pressure in the ion trap region of 2.5 mTorr) have previously been determined to be ca. 3 × 10^9^ molecules cm^−3^ [37]. Each spectrum represents an average of at least 50 scans.

### Ozone-Induced Dissociation

Ozone-induced dissociation (OzID) was performed as previously described [38]. Ozone was generated as described above and collected in a plastic syringe. Ozone was introduced by attaching the plastic syringe to a PEEK-sil tubing restrictor (100 mm L × 1/16′′ o.d. × 0.025 mm i.d.; SGE Analytical Science) connected to the helium supply line via a shut-off ball valve and T-junction. The helium flow rate was controlled using a metering valve. A backing pressure was applied to the syringe (25 μL min^–1^) using a syringe pump, thereby introducing ozone into the ion trap. Ozonolysis reaction time was controlled by adjusting the ion activation time. For phospholipids, the reaction time was 10 s, following which the ion appearing 48 Th above the mass-selected pseudo-molecular ion was isolated and fragmented via CID. For analysis of secondary ozonide decomposition products, the surface-synthesized ozonide was subjected to CID and the product ion resulting from the 46 Da neutral loss was re-isolated in an MS^3^ experiment and allowed to undergo OzID with a reaction time of 1 or 10 s for FAMEs and phospholipids, respectively.

### Photodissociation

Photodissociation (PD) using a Quanta-Ray INDI Nd:YAG pumped optical parametric oscillator (OPO) laser system (Spectra-Physics, Santa Clara, CA, USA) was performed as previously described [39, 40]. Briefly, a quartz viewport was fitted to the back plate of the LTQ chamber to allow transmission of a laser pulse into the vacuum region. Optical access to the ions within the quadrupole ion trap is afforded by the 2 mm orifice centered on the back ion lens. To ensure isolated ions were only activated with a single laser pulse, a mechanical shutter is placed at the exit of the aperture of the OPO and synchronized with the activation sequence of the mass spectrometer using a TTL pulse generated at the beginning of the appropriate MS^n^ activation step. Ions were isolated with a nested isolation sequence of 5, 2, and 10 Da to ensure no fragmentation occurred during application of the isolation waveform. After isolation ions were irradiated using either a 260 or 300 nm laser pulse (~5 ns pulse width at ~1 mJ pulse^–1^) and spectra averaged over 200 scans.

## Results and Discussion

### Decomposition of Secondary Ozonides Formed from Fatty Acid Methyl Esters

**Figure 1:**
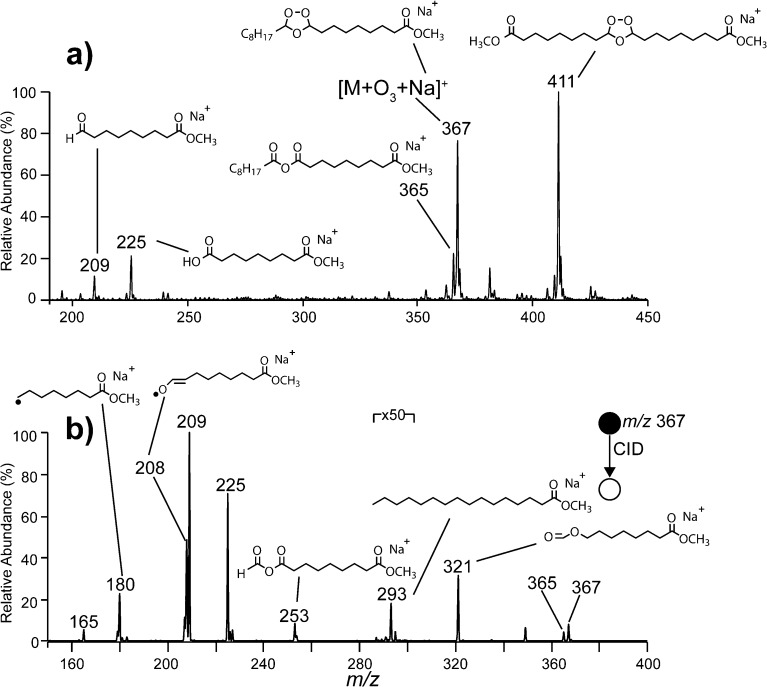
**(a)** Positive ion ESI-MS spectrum acquired from an extract of a thin film of methyl oleate, FAME 9*Z*-18:1, following exposure to ozone. **(b)** The CID mass spectrum obtained from the [M + O_3_ + Na]^+^ ion at *m/z* 367. Assigned structures are indicated

Ozonolysis of a thin film of methyl oleate (FAME 9*Z*-18:1) over several min resulted in complete oxidation and formation of several oxidation products, including secondary ozonides. Figure 1a shows the full ESI-MS spectrum acquired following ozonolysis of FAME 9*Z*-18:1 for 3 minutes and extraction of the reaction products formed on the surface. The [M + Na]^+^ ion expected at *m/z* 319 is absent, confirming complete oxidation, while an abundant ion with a mass 48 Da greater is observed at *m/*z 367 and is assigned as the [M + O_3_ + Na]^+^ ion (i.e., the secondary ozonide). The base peak at *m/z* 411 is assigned to the cross-ozonide formed via the recombination of an aldehyde and carbonyl oxide intermediate from two separate FAME molecules where both fragments contain the methyl ester moiety. This assignment is supported by CID analysis (data not shown) and is consistent with previous literature reports for ozonolysis of simple alkenes, fatty acids (including methyl esters), and triacylglycerols (TAGs) [41–44]. Prior work indicates that cross-ozonide formation is impacted by the concentration of the olefin but this effect was not further investigated here. The ions at *m/z* 209 and 225 observed in the full MS spectrum (Figure 1a) are assigned as an aldehyde (9-oxononanoic acid methyl ester) and carboxylic acid (9-methoxy-9-oxononanoic acid), both of which are well known products of lipid ozonolysis (cf. Scheme 1).

**Scheme 2:**
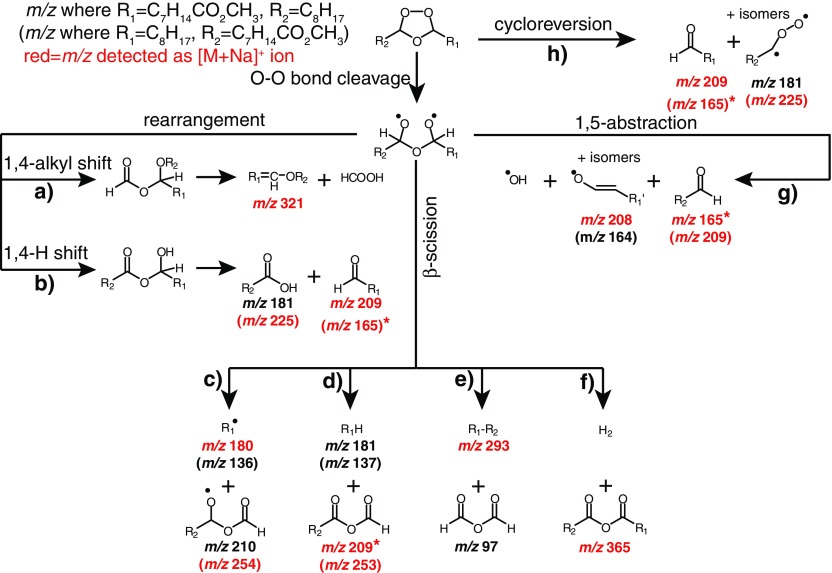
Suggested dissociation pathways of methyl oleate secondary ozonides. Indicated *m/z* values correspond to the expected mass for the corresponding sodium adduct ions. The upper *m/z* values are those expected when R_1_ = C_7_H_14_CO_2_CH_3_ and R_2_ = C_8_H_17_, whereas the *m/z* values in parentheses are expected when R_1_ = C_8_H_17_ and R_2_ = C_7_H_14_CO_2_CH_3_. The *m/z* values shown in red indicate they are observed in the spectrum shown in Figure 1b. In cases where the product contains both R groups, only the one possible *m/z* value is indicated; *m/z* values indicated with an asterisk do not contain the ester moiety and are thus unlikely to be observed. These *m/z* values are most likely dominated by the formation of an isobaric fragment ion containing the ester moiety (e.g., *m/z* 209). An isobaric fragment ion at *m/z* 165 can also form by further dissociation of the *m/z* 180 ion (see main text)

The CID spectrum acquired from the [M + O_3_ + Na]^+^ ion of FAME 9*Z*-18:1 observed at *m/z* 367 is shown in Figure 1b, while Scheme 2 outlines the possible products that can be formed following activation of the secondary ozonide. The *m/z* values highlighted in red are those observed in the CID spectrum in Figure 1b, and the identity and origin of these species are discussed in detail below.

The product ions observed at *m/z* 209 and 225 in Figure 1b correspond in mass to the aldehyde and carbonyl oxide, respectively, although in the latter case, rearrangement of the carbonyl oxide to other isomers is likely (cf. Scheme 1). Given that the structure of the *m/*z 225 ion is ambiguous, we refer to it here (and in similar instances) as the “Criegee ion.” These ions may form via either the biradical mechanism (Scheme 2b) or by cycloreversion of the secondary ozonide (Scheme 2h). Both of these fragments are well known decomposition products of lipid secondary ozonides [20, 34].

The even mass of the *m/z* 180 product ion observed following CID of the [M + O_3_ + Na]^+^ ion in Figure 1b is consistent with a radical species formed by loss of a neutral fragment with the composition, C_10_H_19_O_3_^•^_._ This is likely formed from the initial biradical intermediate following activation of the secondary ozonide through a β-scission mechanism (Scheme 2c). An analogous radical ion has previously been observed following CID of the secondary ozonide formed from the monounsaturated phospholipid 1-palmitoyl-2-oleoyl-*sn*-glycero-3-phosphocholine (PC 16:0/9*Z*-18:1) [34]. If the nascent radical fragment undergoes further reaction within the ion–dipole complex prior to dissociation, analogous β-scission pathways could also account for a number of the other products observed, including (i) the neutral loss of octane to form *m/z* 253 (–114 Da) (Scheme 2d); (ii) loss of formic anhydride to form *m/*z 293 (–74 Da, Scheme 2e), and (iii) loss of dihydrogen to form *m/z* 365 (–2 Da, Scheme 2f). Analysis of the isomeric vaccenic acid methyl ester (FAME 11*Z*-18:1) secondary ozonide gave rise to an analogous suite of product ions (*m/z* 208, 281, 237, and 253) supporting the assignment of these species (Supporting Information, Figure S1).

**Figure 2:**
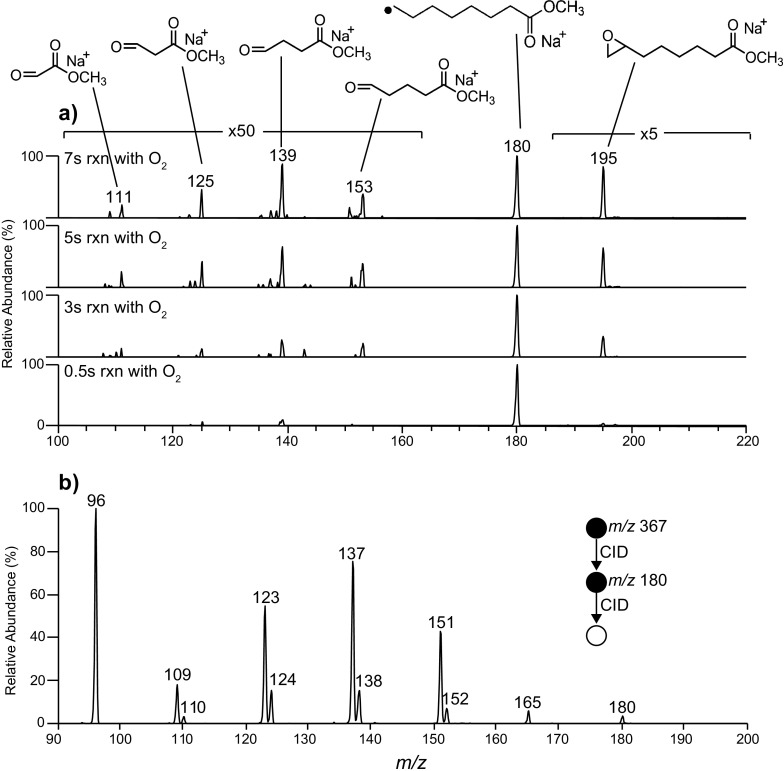
**(a)** Mass spectra resulting from the gas phase reaction of the *m/z* 180 radical ion (formed by CID of the [M + O_3_ + Na]^+^ ion of methyl oleate secondary ozonide) with background dioxygen inside the ion trap with reaction times of 0.5, 3, 5, and 7 s. **(b)** MS^3^ spectrum of the *m/z* 180 radical ion

To confirm the assignment of the *m/z* 180 ion as a carbon-centered radical, this ion was isolated with no additional collisional activation energy applied in an MS^3^ experiment and allowed to react with background oxygen inside the ion trap prior to product ion ejection and mass analysis [37]. It has previously been demonstrated that alkyl radicals readily react with molecular oxygen to form a +32 Da peroxyl radical, which can then undergo secondary decomposition processes that include ejection of a hydroxyl radical to form an epoxide [45, 46]. Thus, observation of similar reactivity provides confirmation for the presence of a carbon-centered radical moiety. Figure 2a shows mass spectra acquired following isolation and trapping of the putative alkyl radical ion in the presence of molecular oxygen over reaction times of 0.5, 3, 5, and 7 s. These spectra reveal a range of product ions arising from the ion-molecule reactions with background dioxygen that increase in abundance with increasing reaction time. The major product formed is the ion at *m/z* 195, which is assigned to an epoxide formed from the peroxyl radical decomposition. The position of the epoxide along the carbon chain could not be uniquely established, and this ion may represent a mixture of epoxide isomers depending of the mechanism(s) of radical migration. Additional fragment ions are observed at *m/z* 111, 125, 139, and 153, and are assigned as carbonyl species formed following other decomposition pathways of the peroxyl radical (or subsequent epoxide) that lead to a series of carbon–carbon bond cleavages. The observation of several ions spaced 14 Da apart is indicative of species differing in mass by a methylene group and suggests that the radical can readily migrate along the carbon chain and thus affect chain-scission at different positions. The CID spectrum acquired from the *m/*z 180 ion (Figure 2b) shows two sets of product ions with characteristic 14 Da spacings that are attributed to a series of dissociation processes that lead to either formation of a shortened alkyl radical by loss of a neutral alkene (even *m/*z values) or, alternatively, ejection of a neutral alkyl radical to produce an alkene-based ion (odd *m/z* values). The base peak at *m/z* 96 is assigned as a methyl acetate radical (i.e., [C_3_H_5_O_2_ + Na]^•+^. The observation of reactions with molecular oxygen and a series of carbon–carbon bond cleavages upon CID is consistent with, and provides strong evidence for, the assignment of the *m/z* 180 fragment as a carbon-centered radical cation.

**Scheme 3:**
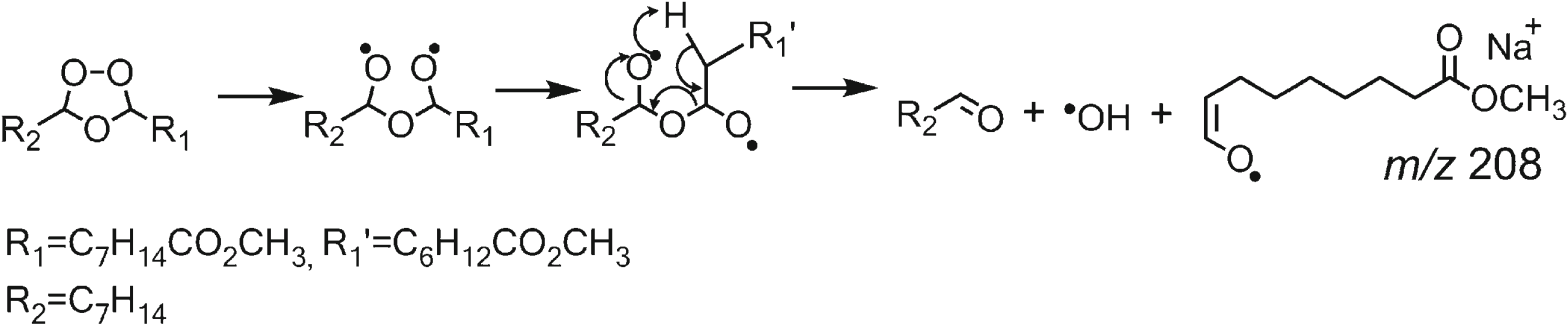
Proposed mechanism for 1,5-hydrogen atom shift within the biradical intermediate to account for the dissociation pathway described in Scheme 2g

The *m/z* 208 product ion observed in Figure 1b is of even mass, suggesting that it too is a radical. This ion is tentatively assigned as an aldehyde enolate radical formed by homolysis of the oxygen–oxygen bond in the parent ozonide, followed by a 1,5-hydrogen atom shift within the biradical intermediate (Scheme 3). Evidence for the enolate-radical structure of *m/z* 208 is provided by its CID spectrum (Supporting Information, Figure S2), which reveals characteristic losses of (i) carbon monoxide to form the *m/z* 180 ion, and (ii) 43 Da (–C_2_H_3_O^•^) to produce *m/z* 165. The latter processes may also contribute to the observation of *m/z* 180 and 165 upon CID of the ozonide [M + O_3_ + Na]^+^ ion (Figure 1b). Further evidence for the assignment of *m/z* 208 as a carbon-centered radical is provided by the observation of its reaction within oxygen when isolated in the ion trap (data not shown). Products of this ion-molecule reaction are similar to those observed for the *m/z* 180 ion (Figure 2a), albeit at lower relative abundance. Interestingly, formation of an abundant product ion at *m/z* 208 via a 1,5-hydrogen atom shift in the biradical intermediate would yield an even-electron aldehyde and a hydroxyl radical as co-products (Scheme 2g). Although the hydroxyl radicals themselves could not be directly detected in this experiment, the observation of other products consistent with this mechanism suggests this pathway may be important for reactive radical production in the atmospheric processing of secondary ozonides.

**Scheme 4:**
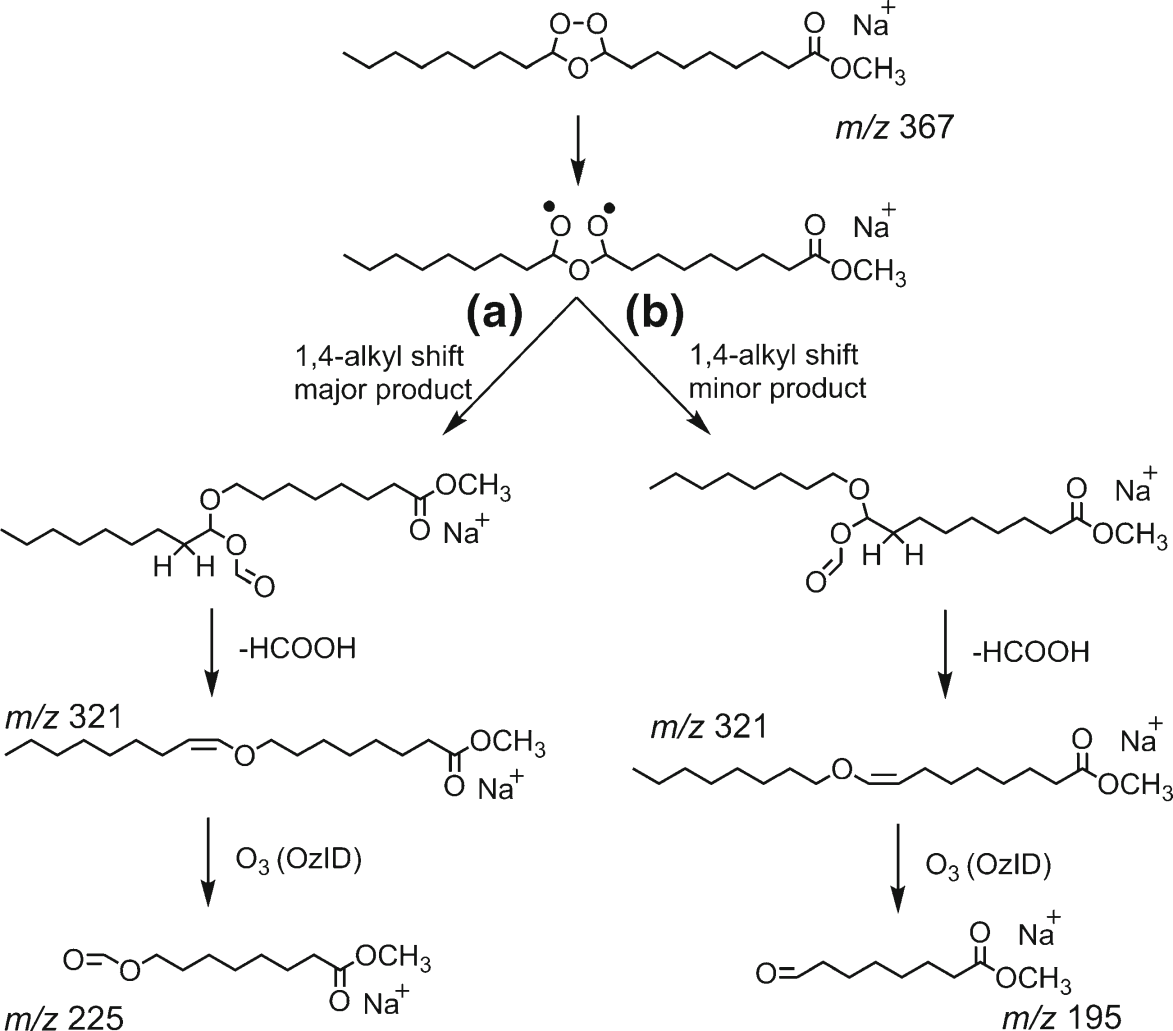
Mechanism for formic acid loss upon CID of methyl oleate secondary ozonide. Mechanism was confirmed by ozone-induced dissociation (OzID) of the corresponding [M+ O_3_ + Na – HCO_2_H]^+^ product ion

The *m/z* 321 ion present in the spectrum shown in Figure 1b is 46 Da lighter than the [M + O_3_ + Na]^+^ precursor ion and is assigned to the loss of formic acid from the secondary ozonide. The loss of formic acid is believed to occur following rearrangement of the biradical by a 1,4-alkyl shift [23] followed by decomposition through *cis*-elimination [47]. The initial alkyl shift can occur in two possible directions (Scheme 4). To characterize the structure of the *m/z* 321 ion and determine the direction in which the alkyl shift occurs, the *m/*z 321 ion was mass-selected and allowed to react with ozone gas leaked into the ion trap to facilitate ozone-induced dissociation [38]. Following a reaction time of 1 s, an abundant product at *m/z* 225 is observed, corresponding to a neutral loss of 96 Da from the [M + O_3_ + Na – HCO_2_H]^+^ ion (Supporting Information, Figure S3). This indicates the new double bond formed upon elimination of formic acid is on the methyl terminus-side of the ozonide, suggesting the dominant formation pathway involves a shift of the alkyl chain attached to the methyl ester terminus and removal of a hydrogen atom from the methyl terminus alkyl chain (cf. Scheme 4a). Interestingly, an ion at *m/z* 195 with a relative intensity ~2% of *m/*z 225 is also observed (Supporting Information, Figure S3) suggesting a small ion population proceeds via the alternative pathway (Scheme 4b). Preference for pathway (a) over (b) is further confirmed by CID of an ozonide formed from *D*_17_-FAME 9*Z*-18:1 in which all hydrogen atoms on the methyl terminus-side of the double bond are labeled (Supporting Information Figure S4). The CID spectrum of the corresponding [M + O_3_ + Na]^+^ ion at *m/z* 384 ion revealed a fragment at *m/z* 337 corresponding to the loss of 47 Da (i.e., loss of CHDO_2_), and confirms removal of a hydrogen from the methyl terminus-end of the ozonide. The mechanism proposed here is consistent with that previously reported by Khachatryan et al. [23] for the gas-phase thermal decomposition of 3,5-dimethyl-1,2,4-trioxolane. The preference for the formic acid loss mechanism to favor one rearrangement direction over the other is likely due to an interaction between the metal ion, ester, and ozonide moieties making one direction energetically preferred. The CID spectrum of *D*_17_-FAME 9*Z*-18:1 also reveals a significant increase in the abundance of *m/z* 226 relative to the non-deuterated case. This potentially provides evidence for the occurrence of Scheme 2d and the observation of an additional *m/z* 209 fragment, where R_2_ = C_8_H_17_ and thus sodium migration from the ester to the anhydride during dissociation. Such charge-exchange within the ion–dipole complexes formed during unimolecular dissociation is well known and can be rationalized in this case by an increase in sodium affinity as the hydrocarbon fragment becomes more heavily oxygenated.

### Decomposition of Secondary Ozonides Formed from Phospholipids

**Figure 3:**
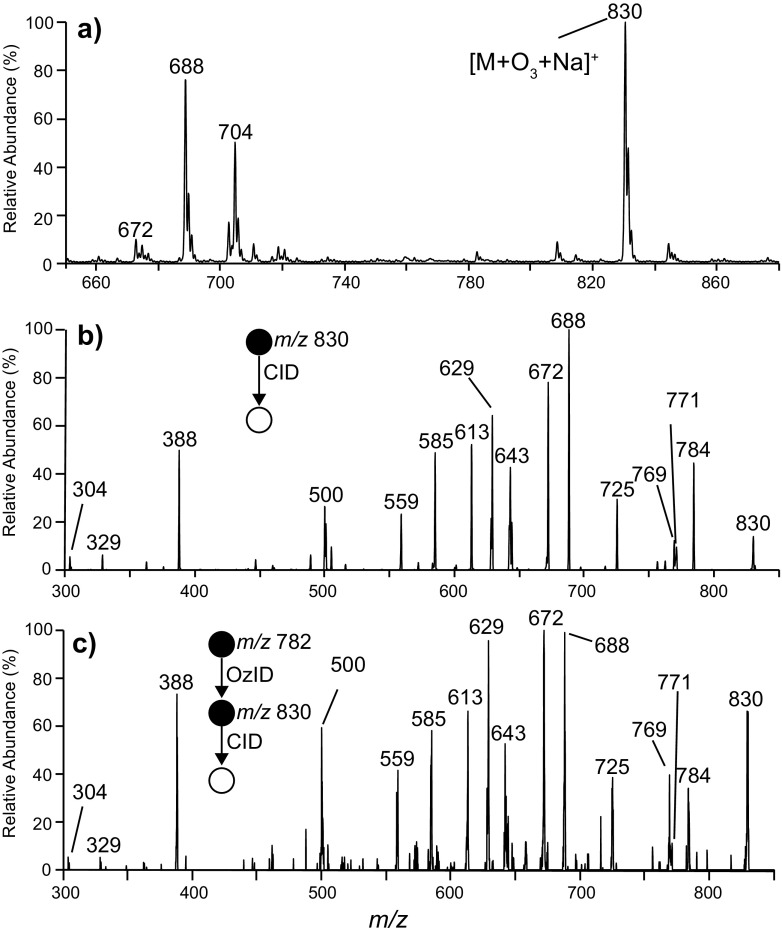
**(a)** ESI-MS spectrum acquired following ozonolysis of a thin film of PC (16:0/9*Z*-18:1) and extraction of reaction products. **(b)** CID spectrum of the [PC (16:0/9*Z*-18:1) + O_3_ + Na]^+^ formed following surface ozonolysis of PC (16:0/9*Z*-18:1). **(c)** CID spectrum of [PC (16:0/9*Z*-18:1) + Na + O_3_]^+^ formed following the gas-phase reaction of ionized [PC (16:0/9*Z*-18:1) + Na]^+^ with ozone

Phospholipids form the major components of cellular membranes in most organisms and are thus likely components of organic aerosols. Ozonolysis of a thin film of the common phosphatidylcholine, PC 16:0/9*Z*-18:1, was conducted and the reaction products extracted from the surface and analyzed by ESI-MS. The resulting positive ion mass spectrum (Figure 3a) shows a base peak at *m/z* 830 that can be assigned as the [M + O_3_ + Na]^+^ ion (*i.e.,* the secondary ozonide) whereas the low abundance ion at *m/z* 782 corresponds to the [M + Na]^+^ ion from unreacted phospholipid. The ions at *m/z* 672 and 688 are assigned as the aldehyde and carboxylic acid ions (or an isomer thereof), respectively. These ions are formed from cleavage of the *n-9* double bond and are analogous to those observed for FAME 9*Z*-18:1 (cf. Figure 1a). The *m/z* 704 ion is attributed to a hemiacetal formed by addition of methanol from the ESI solvent to the aldehyde ion. Evidence for this structure is provided by CID, which reveals an abundant loss of methanol (–32 Da) to form the parent aldehyde (data not shown) and is consistent with previous observations [18].

The CID spectrum of the phospholipid [M + O_3_ + Na]^+^ ion at *m/z* 830 is shown in Figure 3b. In this spectrum, the *m/z* 771 product ion arises from loss of trimethylamine from the phosphocholine head group (–59 Da), a characteristic fragment arising from activation of sodiated PCs [48, 49]. The *m/z* 769 ion is assigned to the subsequent loss of dihydrogen from the [M + O_3_ + Na – N(CH_3_)_3_]^+^ ion and likely has an anhydride structure analogous to that assigned for decomposition of the FAME 9*Z*-18:1 ozonide (cf. Scheme 2f). The product ions at *m/z* 672 and 688 are assigned as the aldehyde and Criegee ion [18, 34, 38], whereas the *m/z* 613 and 629 ions correspondingly arise from further loss of trimethylamine from these species. Formic acid loss is also observed from the PC (16:0/9*Z*-18:1) ozonide resulting in a product ion at *m/z* 784. Further interrogation of the structure of this ion by OzID revealed an abundant loss of 96 Da (Supporting Information, Figure S5), thereby confirming formic acid loss occurs via a mechanism identical to that described for FAME 9*Z*-18:1 ozonide (Scheme 4).

**Figure 4:**
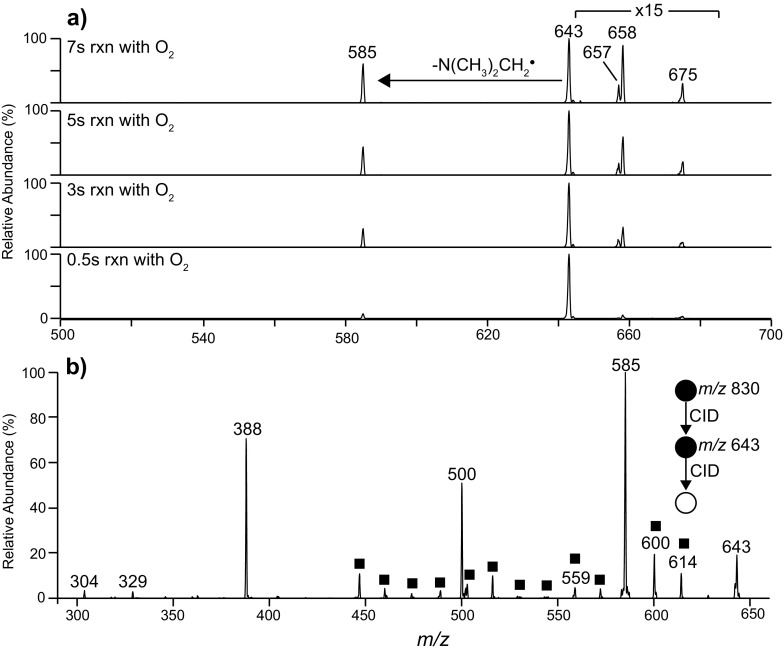
**(a)** Reaction of the *m/z* 643 radical formed by CID of the [M + O_3_ + Na]^+^ ion formed from PC (16:0/9*Z*-18:1) with background dioxygen inside the ion trap with reaction times of 500 ms, 3 , 5, and 7 s. **(b)** CID spectrum of the *m/z* 643 radical formed as described in **(a)**. ■ = fragments arising from carbon-carbon β-scission along the alkyl chains

Carbon-centered radicals were also observed from CID of the ionized PC (16:0/9Z-18:1) ozonide. The *m/z* 643 ion (Figure 3b) is assigned as an alkyl radical formed via an identical process to the *m/z* 180 ion produced from the FAME 9*Z*-18:1 ozonide (cf. Scheme 2c). Harrison and Murphy observed the analogous radical following CID of the [M + O_3_ + H]^+^ ion derived from ozonolysis of the same PC [34]. To confirm the assignment of *m/*z 643 as a carbon-centered radical, the ion was isolated and allowed to react with background oxygen within the ion trap (Figure 4a). In this experiment, ions at *m/z* 675 (+32 Da) and 658 (+15 Da) were observed to increase in abundance with increasing reaction times, and are thus assigned as a peroxyl radical and an epoxide, respectively. The observation of the peroxyl radical for phosphatidylcholine but not for the FAME is explained by the larger size of the phospholipid harboring more vibrational degrees of freedom that stabilizes the adduct upon the exothermic addition of dioxygen. This reactivity of the *m/z* 643 ion towards molecular oxygen is consistent with its assignment as a carbon-centered radical. Interestingly, the most abundant product ion formed upon the reaction of *m/z* 643 with O_2_ appears at *m/z* 585 (Figure 4a). This corresponds to a loss of 58 Da, suggesting ejection of N(CH_3_)_2_(CH_2_)^•^ following intramolecular hydrogen atom abstraction and radical migration to the phosphocholine head group. Analogous head group decompositions were observed for dioxygen reactions with ozonide-derived radical ions from phosphatidylglycerol (PG) (–73 Da), phosphatidylserine (PS) (–86 Da), and phosphatidylethanolamine (PE) (–42 Da) (Supporting Information, Figure S6). The observation of radical reactivity arising remote from the initial ozonide site is indicative of radical migration towards more stabilized positions.

**Scheme 5:**
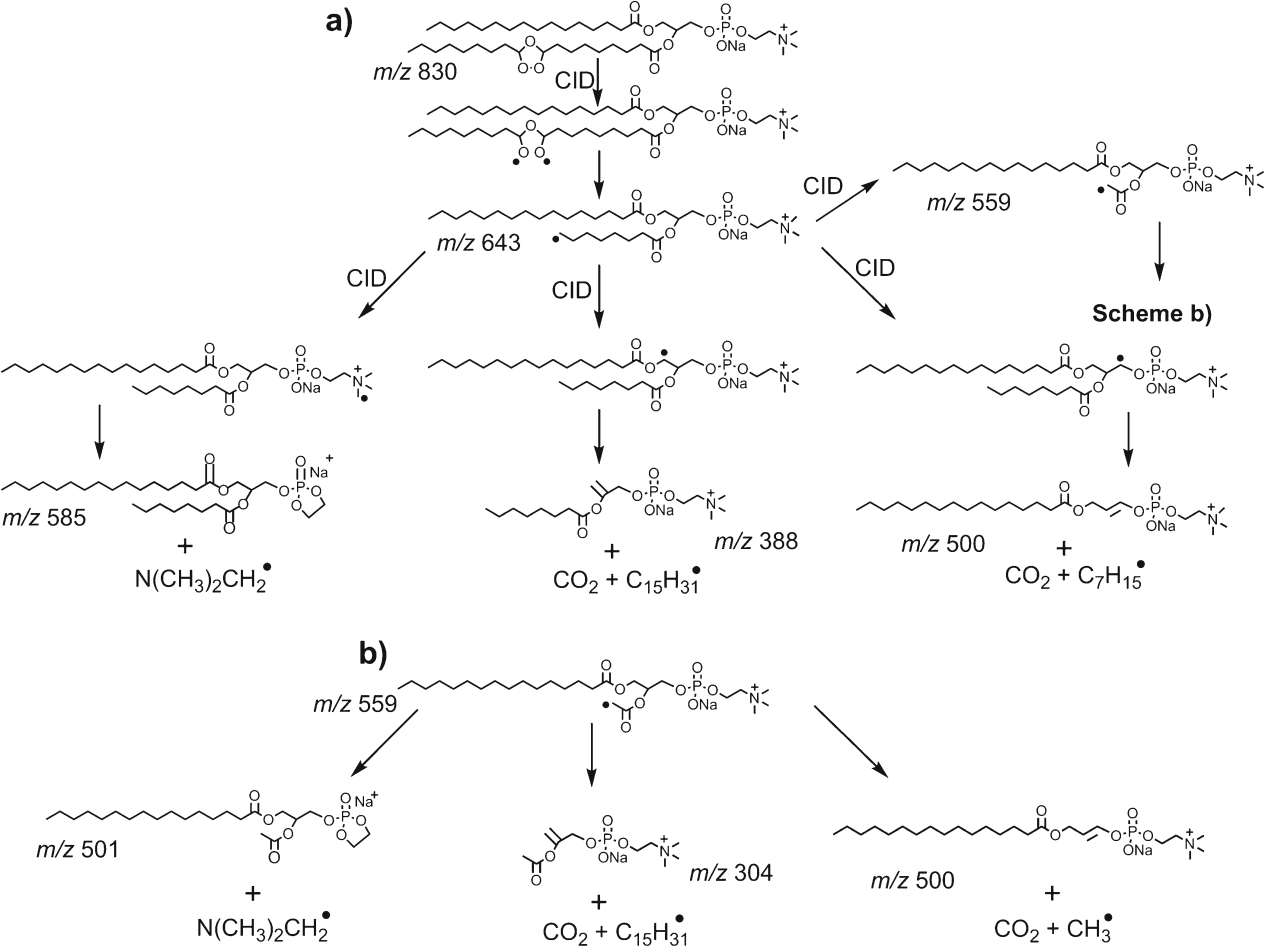
**(a)** Biradical-driven decomposition pathways of the [PC (16:0/9*Z*-18:1) + O_3_ + Na]^+^ secondary ozonide upon CID; **(b)** indicates the further decomposition pathways of the *m/z* 559 product ion

Figure 4b shows the CID spectrum obtained from the *m/z* 643 radical ion and provides further evidence for radical migration as outlined in Scheme 5. The base peak at *m/z* 585 corresponds to a neutral loss of 58 Da and again is assigned as the loss of N(CH_3_)_2_CH_2_^•^. Analogous losses are also observed following CID of the corresponding radicals formed from ionized PG (see below), PS, and PE secondary ozonides (data not shown). The product ion at *m/z* 388 in the spectrum shown in Figure 4b corresponds to a loss of 255 Da and is consistent with neutral radical loss of the constituents of the palmitic acid (16:0) moiety. This observation is evidence for radical migration to the glycerol backbone followed by ejection of CO_2_ and C_15_H_31_^•^ (Scheme 5). Further evidence supporting this pathway is provided by an identical analysis of 1-stearoyl-2-oleoyl-*sn*-glycero-3-phosphocholine secondary ozonide (i.e., [PC (18:0/9*Z*-18:1) + O_3_ + Na]^+^. Upon CID of the equivalent *m/*z 671 radical, a neutral loss of 283 Da is observed, indicating loss of stearic acid (18:0) as CO_2_ and C_17_H_35_^•^ (Supporting Information, Figure S7). Alternatively, radical migration to the adjacent glycerol carbon leads to the loss of the truncated fatty acid chain by ejection of CO_2_ and C_7_H_15_^•^ and can account for formation the *m/*z 500 product ion (Scheme 5). An additional series of low abundance ions spaced by 14 Da are also observed (indicated with a square symbol in Figure 4b). This product ion sequence is indicative of consecutive β-cleavages along the alkyl chains. Interestingly, the observation of this homologous series of product ions extending below *m/z* 500 points to cleavages along the 16:0 fatty acid and serves as evidence for radical migration to the adjacent saturated acyl chain.

The radical ion observed at *m/*z 559 in Figure 4b can arise from radical migration and carbon–carbon bond cleavage yielding an acetate-like radical analogous to the *m/*z 96 ion formed from FAME 9*Z*-18:1 (Figure 2b). Further evidence for this assignment is the observation of a similar reactivity towards molecular oxygen as that observed for *m/z* 643 (data not shown) and its prior observation as a CID product of the corresponding [M + O_3_ + H]^+^ ion [34]. This radical cation can then undergo decomposition via radical migration to produce the fragment ions at *m/z* 304, 500, and 501 (Supporting Information, Figure S8).

### PD of Ozonides

**Figure 5:**
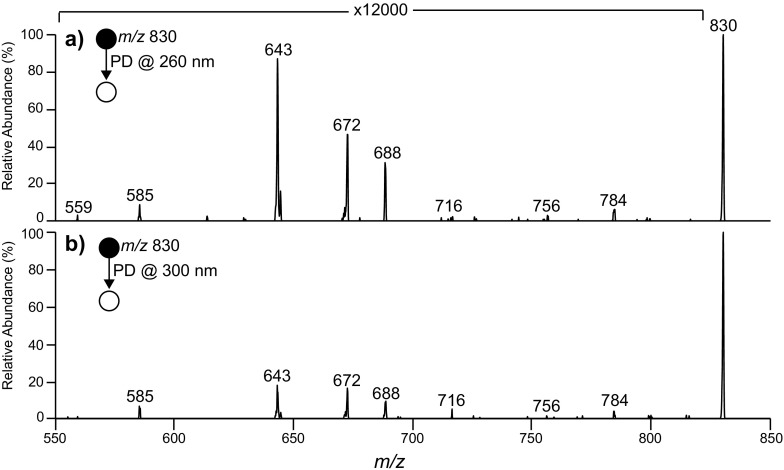
PD spectra acquired from the secondary ozonide ion, [M + O_3_ + Na]^+^, of PC (16:0/9*Z*-18:1) at **(a)** 260 nm and **(b)** 300 nm

To investigate the PD of lipid ozonides that could be of potential relevance the atmosphere [50], surface-synthesized ozonides were extracted and ionized by electrospray ionization. The resulting [M + O_3_ + Na]^+^ cations were mass-selected and isolated inside the ion trap mass spectrometer before being subjected to UV irradiation. Figure 5 shows the UV-PD spectra acquired at either 260 or 300 nm from the ionized secondary ozonide of PC (16:0/9*Z*-18:1). To control for dissociation of the ozonide during mass selection of the precursor, a nested isolation protocol was employed (see Experimental section for details) and under these conditions no background dissociation was observed in the absence of laser irradiation (data not shown). The large magnifications required to visualize the PD product ions in Figure 5 provide evidence of the low yield of these photochemical reactions, which is largely attributed to the poor absorption of lipid ozonides at these wavelengths as previously shown [51]. Despite the low yield, however, the PD spectra in Figure 5 clearly indicate that UV activation leads to the decomposition of the ionized secondary ozonide yielding many of the identical products to those observed from CID (Figure 3b) albeit at much lower abundance.

The most abundant PD product ions in the spectra in Figure 5 can be assigned as aldehyde (*m/z* 672), Criegee (*m/z* 688), and alkyl radical (*m/z* 643) ions. Additional product ions observed at lower abundance include *m/z* 784, 756, and 716, and are assigned to losses of formic acid, formic anhydride, and octane, respectively [24, 31]. These product channels are attributed to decomposition via the biradical intermediate (cf. Scheme 2). Furthermore, lower mass ions at *m/z* 585 and 559 are detected and assigned to decomposition of alkyl radicals following radical migration (Scheme 5). It is interesting to note that ions at *m/*z 756 and 716 are also observed in the corresponding CID spectrum (cf. Figure 3b) but at relatively low abundance. Another key comparison is that in the λ = 260 nm PD spectrum (Figure 5a), the abundance of odd-electron products relative to the combined abundance of the even-electron (e.g., aldehyde and Criegee ions) is higher than that observed from CID (Figure 3b). One possible explanation is an electronic excited state process that delivers selectivity for oxygen–oxygen scission (and thus radical-driven dissociation) over competing even-electron dissociation upon photoactivation. Further evidence for this is the absence of any product ions in the PD spectrum not associated with ozonide decomposition (e.g., loss of N(CH_3_)_3_). CID presumably occurs via electronic ground state pathways whereas UV photoexcitation accesses electronically excited states. Whether dissociation following UV photolysis occurs directly from excited electronic states or on the electronic ground state following state-crossing processes is not known but an intriguing question for further study.

### Comparison of Ozonides Formed on Surfaces Versus the Gas Phase

The ability to observe gas-phase ozonolysis of ionized lipids within an ion trap provides a unique opportunity to directly compare surface-formed secondary ozonides (discussed above) with their gas-phase counterparts. It is generally accepted that secondary ozonides are not formed to a significant extent in the gas phase because of the lack of stabilization of the vibrationally excited carbonyl oxide. This ultimately results in unimolecular decomposition and/or rearrangement before any recombination with the partnering carbonyl compound can occur [3]. Despite this, under certain conditions several studies have detected secondary ozonides from the ozonolysis of small alkenes in the gas phase [52–54]. However, the gas-phase secondary ozonide formation for larger lipid molecules has not been investigated.

**Figure 6:**
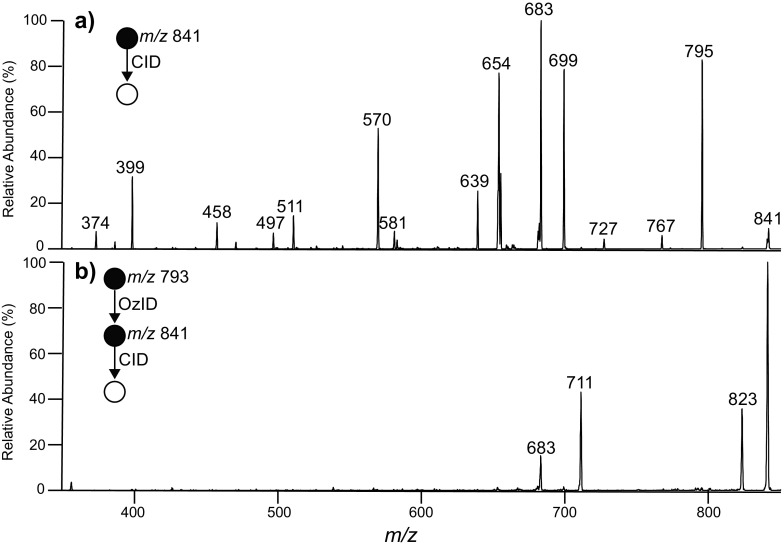
**(a)** CID) spectrum of the [PG (16:0/9*Z*-18:1) + O_3_ + 2Na – H]^+^ ion formed by surface ozonolysis of PG (16:0/9*Z*-18:1) and subsequent ESI-MS analysis. **(b)** CID spectrum of [PG (16:0/9*Z*-18:1) + 2Na – H + O_3_]^+^ formed in the gas phase following ozone-induced dissociation (OzID) to induce gas-phase ozonolysis of [PG (16:0/9*Z*-18:1) + 2Na - H]^+^

The CID spectrum acquired from the [M + Na + O_3_]^+^ ion formed following gas-phase ozonolysis of the unsaturated PC (16:0/9*Z*-18:1) is shown in Figure 3c. Although the signal-to-noise of this spectrum is low, there is remarkable similarity with the CID spectrum of the [M + O_3_ + Na]^+^ secondary ozonide ion arising from surface ozonolysis (Figure 3b)_._ This similarity provides evidence for formation of a population of stable secondary ozonides during gas-phase ozonolysis of the ionized lipid. Congruent CID spectra were also obtained following gas-phase and surface-prepared ozonides from triacylglycerols (e.g., TAG (18:0/16:0/9*Z*-18:1, data not shown). In contrast, Figure 6a shows the CID spectra acquired from the [M + O_3_ + 2Na – H]^+^ of the phosphatidylglycerol PG (16:0/9*Z*-18:1) following surface ozonolysis and the corresponding [M + O_3_+ 2Na – H]^+^ ion resulting from gas-phase ozonolysis (Figure 6b). CID of the surface-formed ozonide reveals many equivalent product ions to those observed for the analogous PC, (cf. Figure 3b). For example, the ions at *m/z* 581 and 487 arise from the different head group structure and correspond to the head group loss of C_3_H_5_O_2_^•^ (–73 Da) from the corresponding carbon-centered radicals at *m/*z 654 and 570, respectively. Thus, the fragmentation behavior is consistent with a secondary ozonide structure. In contrast, CID of the [M + O_3_ + 2Na – H]^+^ ion formed in the gas-phase ion-molecule reaction yields different product ions and thus points to a structural isomer (Figure 6b). Comparative analyses of gas phase- and surface-formed ozonides of unsaturated PS and PE lipids also reveal extensive spectral differences (data not shown). One explanation for these observations is that phospholipids containing nucleophilic moieties such as the hydroxyl and amino groups present in the head groups of PG, PS, and PE may be capable of interacting with the initial primary ozonide to facilitate isomerization that outcompetes secondary ozonide formation. In contrast, lipids without a nucleophilic head group (i.e., PC and triacylglycerols) are capable of forming stable secondary ozonides in the gas-phase. Importantly, the results obtained for PC provide the first demonstration of the formation of stable of phospholipid secondary ozonides from gas-phase ozonolysis.

## Conclusions

Ion-trap mass spectrometry has provided direct evidence for the products arising from the unimolecular dissociation of lipid secondary ozonides of the type likely present on the surface of both marine and other aerosols. These findings complement recent evidence for the formation of secondary ozonides upon reaction of lipid-based aerosols with ozone [55, 56]. The unequivocal demonstration that carbon-centered radicals are formed upon both collisional- and photo-activation of these lipid ozonides indicates that these processes may need to be considered as pathways for surface modification of aerosols in the troposphere. Interestingly, some of the mechanisms proposed to account for radical-driven dissociation of lipid ozonides posit hydroxyl radical as a co-product. The release of hydroxyl radicals during decomposition of lipid secondary ozonides represents an important avenue for chain propagation of free radical oxidation. Furthermore, many other products of ozonolysis (such as aldehydes and ketones) can open additional pathways to photolysis and free radical production, and may initiate further reactions and surface modifications [50].

Finally, mechanisms for radical production from activation of lipid secondary ozonides all proceed from initial homolysis of the oxygen–oxygen bond and subsequent dissociation and rearrangement of the resulting biradical intermediate. Recent studies suggest that similar ozonolysis mechanisms in polymers proceed via surface-crossing from the singlet to the triplet biradical surfaces [57]. Future identification of minimum energy crossing points for lipid biradicals may provide insight into the competition between even- and odd-electron processes in lipid ozonide decomposition.

## Electronic supplementary material

Below is the link to the electronic supplementary material.

ESM 1 (PDF 293 kb)
