# Supplementary material for: Radical Generation from the Gas-Phase Activation of Ionized Lipid Ozonides
Source: J Am Soc Mass Spectrom. 2017 May 8;28(7):1345–58. doi: 10.1007/s13361-017-1649-4 (PMC5486690; doi:10.1007/s13361-017-1649-4)
Supplement: Supplementary file 1 — (PDF 293 kb) [file 13361_2017_1649_MOESM1_ESM.pdf]

SUPPORTING INFORMATION

for

Radical generation from the gas-phase activation of ionized lipid ozonides

Shane R. Ellis*[a,b], Huong T. Pham[b†], Adam J. Trevitt[b], Todd W. Mitchell[c], Marc in het Panhuis[b] and Stephen J. Blanksby[d]*

[a] M4I, The Maastricht Multimodal Molecular Imaging Institute, University of Maastricht, 6229 ER Maastricht, The Netherlands

[b] School of Chemistry, University of Wollongong, Wollongong, NSW 2522, Australia

[c] School of Medicine, University of Wollongong, Wollongong, NSW 2522, Australia

[d] Central Analytical Research Facility, Institute for Future Environments, Queensland University of Technology, Brisbane, QLD 4001, Australia

* Authors to whom correspondence should be addressed:

s.ellis@maastrichtuniversity.nl

stephen.blanksby@qut.edu.au

[†] Present address:

Boreal Ecosystem Research Initiative (BERI), Environmental Science, Grenfell campus, Memorial University, Corner Brook, NL A2H 5G4, Canada

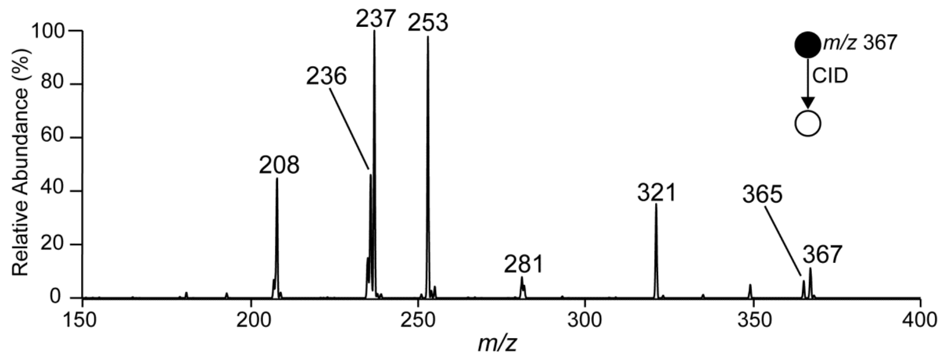

Figure S1. CID spectrum of the secondary ozonide $[M+Na+O_3]^+$ ion formed from surface ozonolysis methyl vaccenate and subsequent ESI-MS analysis.

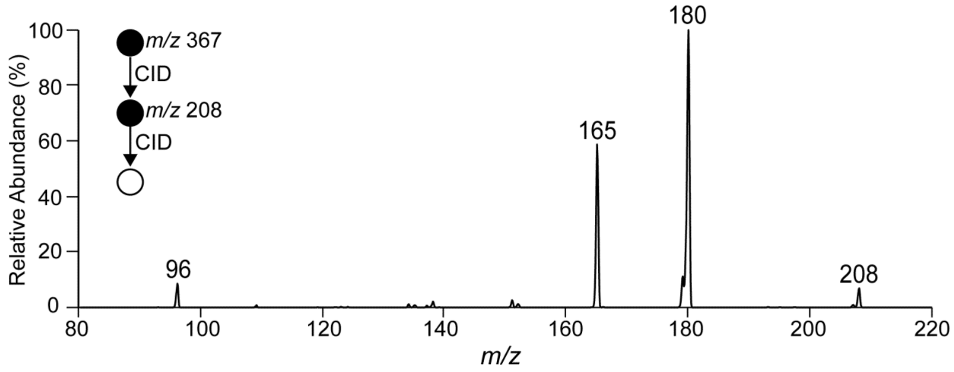

Figure S2. MS^3 CID spectrum of the *m/z* 208 radical formed by CID of $[M+O_3+Na]^+$ ion formed by surface ozonolysis of methyl oleate (FAME 9*Z*-18:1) and ESI-MS analysis.

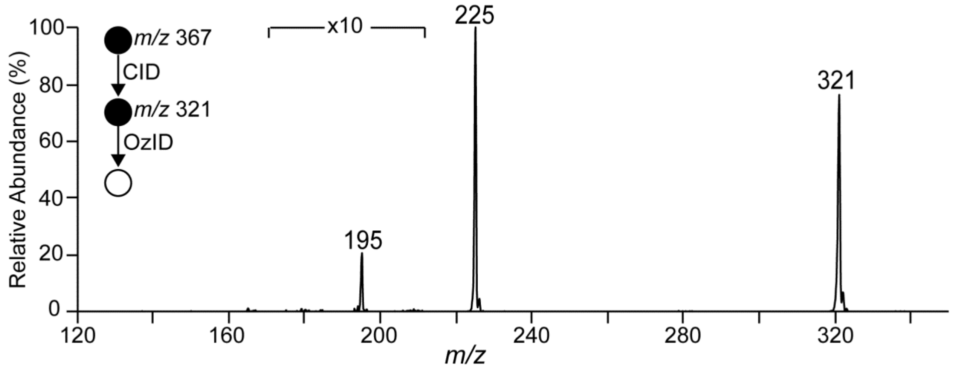

Figure S3. CID/OzID spectrum acquired by OzID of the *m/z* 321 product ion produced from CID of the $[M+O_3+Na]^+$ ion of methyl oleate (FAME (9*Z*-18:1).

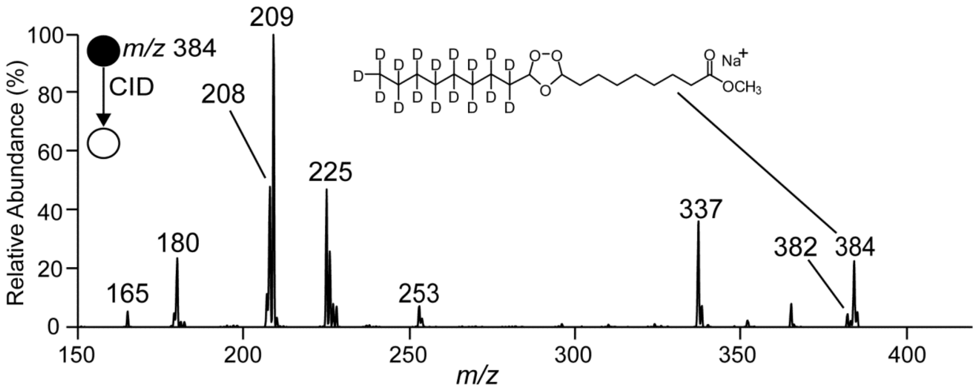

Figure S4. CID spectrum of the secondary ozonide $[M+O_3+Na]^+$ ion formed from ozonolysis of D_{17}-methyl oleate (FAME 9*Z*-18:1 and subsequent ESI-MS analysis.

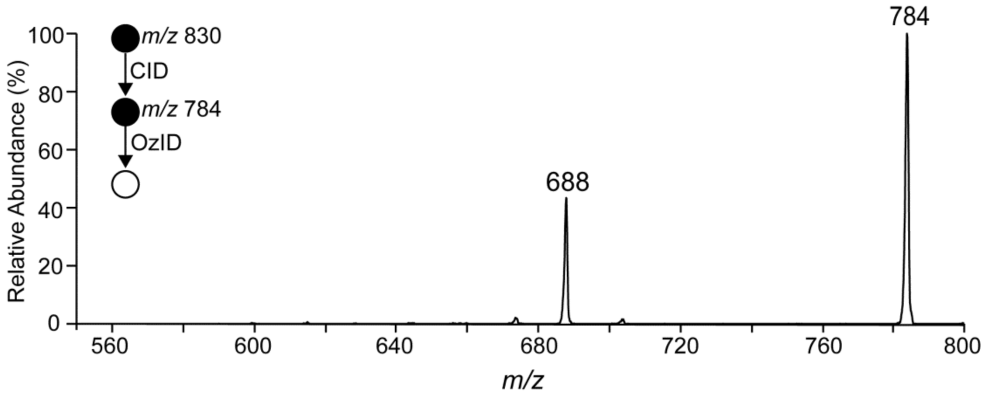

Figure S5. CID/OzID spectrum acquired by OzID of the *m/z* 784 product ion produced from CID of the $[M+O_3+Na]^+$ ion of PC (16:0/9*Z*-18:1).

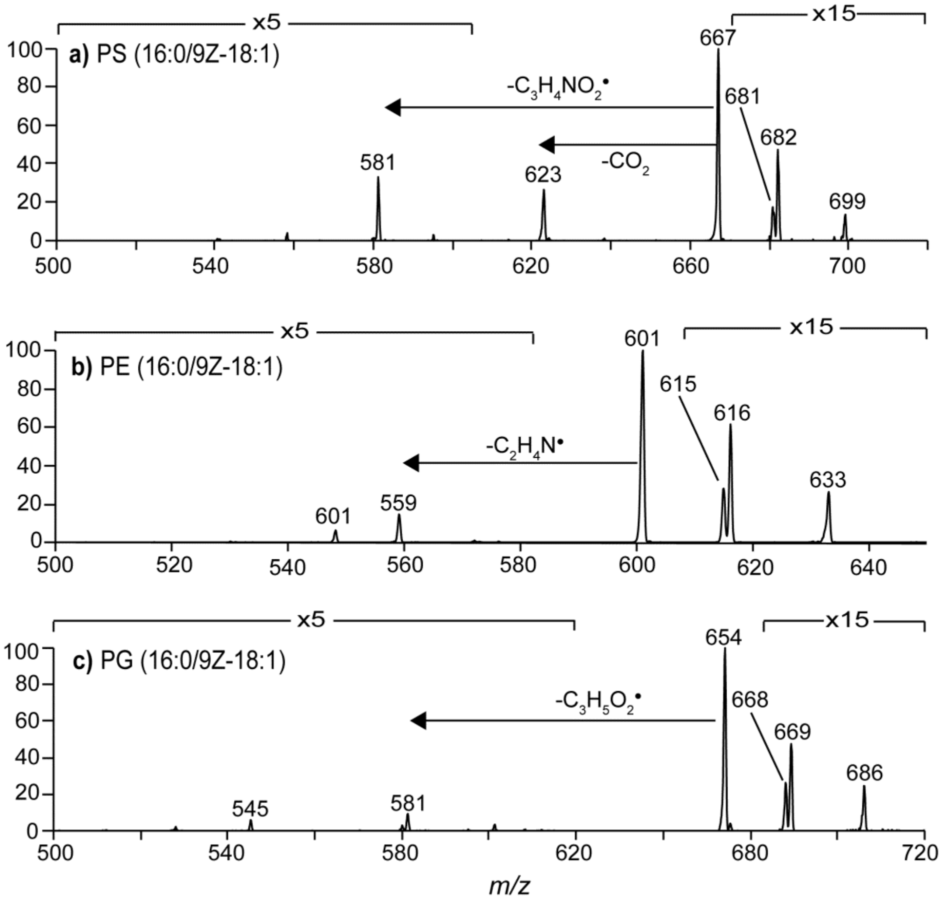

Figure S6. Spectra acquired following a 7 s reaction of background dioxygen inside the ion trap with the radical product ions (a) [(PS (16:0/9*Z*-18:1)+O_3+2Na-H-$C_{10}H_{19}O_3^{\bullet}$]$^{+\bullet}$, (b) [(PE (16:0/9*Z*-18:1)+O_3+Na-$C_{10}H_{19}O_3^{\bullet}$]$^{+\bullet}$, and (c) [(PG (16:0/9*Z*-18:1)+O_3+2Na-H-$C_{10}H_{19}O_3^{\bullet}$]$^{+\bullet}$ formed following CID of the corresponding secondary ozonides. All lipids undergo radical loss of the polar headgroup following reaction with dioxygen and radical migration.

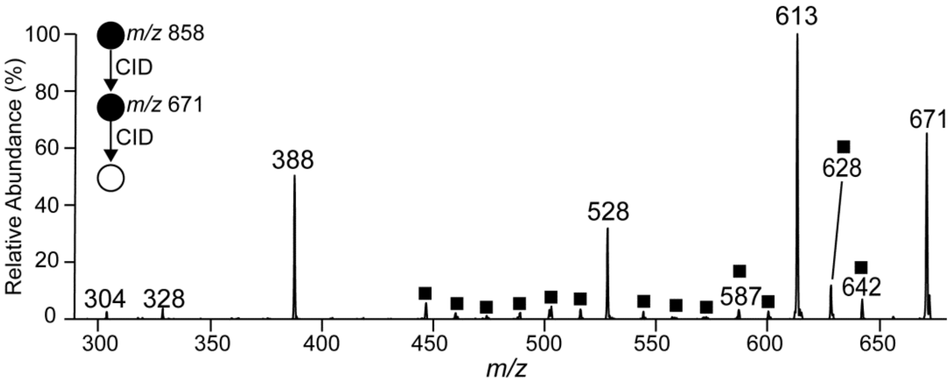

Figure S7. CID spectrum of the *m/z* 671 radical cation formed by CID of the secondary ozonide [M+O_3+Na]$^+$ ion produced from by surface ozonolysis of PC (16:0/9*Z*-18:1). ■=fragments arising from carbon-carbon β-cleavages along the alkyl chains.

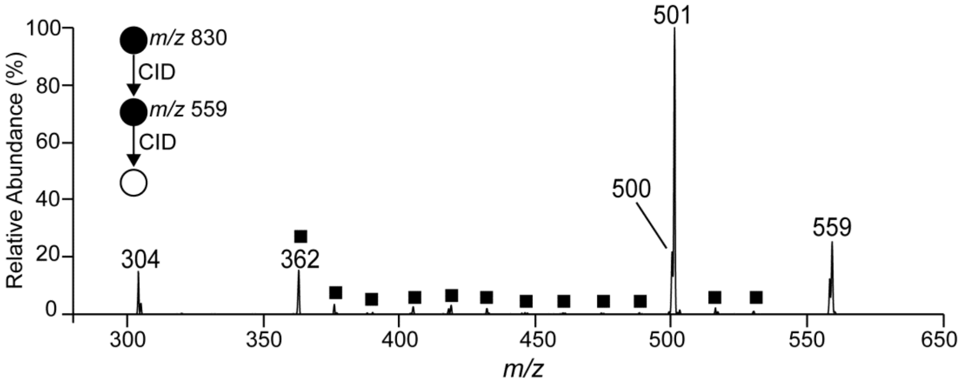

Figure S8. CID spectrum of the *m/z* 559 radical cation by CID of the secondary ozonide $[M+O_3+Na]^+$ ion produced by surface ozonolysis of PC (16:0/9*Z*-18:1) ■ = fragments arising from carbon-carbon β-cleavages along the alkyl chains. Note that it is also possible that some *m/z* 500 ion arises from activation of the small amount of *m/z* 558 isolated upon mass-selection of *m/z* 559.
